# Heterologous expression and biochemical characterisation of the recombinant β-carbonic anhydrase (MpaCA) from the warm-blooded vertebrate pathogen *malassezia pachydermatis*

**DOI:** 10.1080/14756366.2021.1994559

**Published:** 2021-12-11

**Authors:** Viviana De Luca, Andrea Angeli, Valeria Mazzone, Claudia Adelfio, Vincenzo Carginale, Andrea Scaloni, Fabrizio Carta, Silvia Selleri, Claudiu T. Supuran, Clemente Capasso

**Affiliations:** a Institute of Biosciences and Bioresources, CNR, Napoli, Italy; b Proteomics & Mass Spectrometry Laboratory, ISPAAM, CNR, Naples, Italy; c Department of Neurofarba, Section of Pharmaceutical and Nutraceutical Sciences, University of Florence, Sesto Fiorentino, Italy

**Keywords:** *Malassezia pachydermatis*, carbonic anhydrase, CO_2_-sensing, bicarbonate, protonography, kinetic parameters, sulphonamides

## Abstract

Warm-blooded animals may have *Malassezia pachydermatis* on healthy skin, but changes in the skin microenvironment or host defences induce this opportunistic commensal to become pathogenic. Malassezia infections in humans and animals are commonly treated with azole antifungals. Fungistatic treatments, together with their long-term use, contribute to the selection and the establishment of drug-resistant fungi. To counteract this rising problem, researchers must find new antifungal drugs and enhance drug resistance management strategies. Cyclic adenosine monophosphate, adenylyl cyclase, and bicarbonate have been found to promote fungal virulence, adhesion, hydrolase synthesis, and host cell death. The CO_2_/HCO_3_^-^/pH-sensing in fungi is triggered by HCO_3_^-^ produced by metalloenzymes carbonic anhydrases (CAs, EC 4.2.1.1). It has been demonstrated that the growth of *M. globosa* can be inhibited *in vivo* by primary sulphonamides, which are the typical CA inhibitors. Here, we report the cloning, purification, and characterisation of the *β*-CA (MpaCA) from the pathogenic fungus *M. pachydermatis*, which is homologous to the enzyme encoded in the genome of *M. globosa* and *M. restricta*, that are responsible for dandruff and seborrhoeic dermatitis. Fungal CAs could be thus considered a new pharmacological target for combating fungal infections and drug resistance developed by most fungi to the already used drugs.

## 1. Introduction

*Malassezia pachydermatis*, originally named *Pityrosporum pachydermatis* since isolated from the scales of an Indian rhinoceros (*Rhinoceros unicornis*) with exfoliative dermatitis, is one of the cutaneous commensals in all warm-blooded animals^1^^,^^2^. This opportunistic commensal has the potential to become a pathogen if the skin microenvironment or host defences are altered^1^. *M. pachydermatis* relevance has been recognised in both veterinary and human medicine^3^. Generally, *M. pachydermatis* is related to otitis externa and seborrhoeic dermatitis in dogs, cats, and wild animals^3^. Although the Malassezia species, such as *M. furfur*, *M. sympodialis*, *M. globosa*, *M. obtusa*, *M. restricta* and *M. slooffiae* are lipid dependent, *M. pachydermatis* is the only species that does not require lipids for its growth^1^^,^^2^. Human skin is commonly colonised by lipid-dependent Malassezia yeasts but rarely by *M. pachydermatis*^4^. However, *M. pachydermatis*, together with *M. furfur* and *M. sympodialis*, have been isolated from bloodstream infections, contributing to fungemia in hospitalised and severely immunocompromised patients, such as preterm neonates, cancer patients, or patients with pulmonary distress^4^.

Azoles and the polyene amphotericin B (AmB) are often used to treat Malassezia-related illnesses in humans and animals^4^^,^^5^. Topical antifungal medications, mainly azole compounds, can effectively treat localised skin lesions. Still, severe cutaneous problems or fungal infections in the lung spreading throughout the whole body can require the use of triazoles drugs, like itraconazole (ITZ) or fluconazole (FLZ)^4^^,^^5^. Unfortunately, the fungistatic properties of azoles and their derivatives, coupled with the continuous usage in the treatment of fungal infections, aided in selecting and establishing drug-resistant fungus strains^5^^,^^6^. The discovery of novel antifungal medications and the improvement of therapeutic strategies to combat drug resistance are required to address and overcome this challenge.

Intriguing, pathogenic and opportunistic fungi can sense changes in the environmental CO_2_ levels, which influence the fungal virulence or their environmental survival fitness^6^^,^^7^^,^^8^. As a result, the fungal CO_2_-sensing represents a promising target for drugs since controlling fungal differentiation and expression of proteins required for virulent or non-virulent qualities may be pharmacologically relevant. As demonstrated in many fungi, the fungal CO_2_-sensing is governed by bicarbonate (HCO_3_^-^), which promotes meiosis and sporulation^9^; and by adenylyl cyclase (AC) as well as cyclic adenosine monophosphate (cAMP), which are involved in spore production^6^^,^^10^^,^^11^. In *Candida albicans*, AC, cAMP, and HCO_3_^-^ have been shown to stimulate filamentous structures (hyphae) needed for fungal virulence, adhesion, production of hydrolases, and inducing cell death in the hosts^6^^,^^12^^,^^13^. Thus, AC, cAMP signalling, and CO_2_/HCO_3_^-^ sensing were suggested as essential elements modulating fungal metabolism and pathogenicity^6^.

The fungal CO_2_-sensing, related to the CO_2_/HCO_3_^−^/pH-sensing, is directly triggered by HCO_3_^−^ generated from the action of metalloenzymes known as carbonic anhydrases (CAs, EC 4.2.1.1). CAs represent a superfamily of ubiquitous enzymes catalysing a fundamental reaction for all living organisms, the reversible hydration of CO_2_ to HCO_3_^−^ and H^+^ (CO_2_ + H_2_O ⇋ HCO_3_^−^ + H^+^)^14–20^. Up to date, eight CA gene families or classes have been identified and named with the letters of the Greek alphabet (α, β, γ, δ, ζ, η, θ, ι)^14–18^. In the fungal kingdom, the typical class is represented by *β*-CAs, and most fungi encode at least one member of this subfamily of enzymes^11^^,^^21^^,^^22^. In contrast, in some filamentous ascomycetes it is possible to find genes also encoding for *β*-CAs^11^^,^^21^^,^^22^.

It has been demonstrated that primary sulphonamides, typical CA inhibitors (CAIs), can inhibit the growth of *M. globosa in vivo* when the CO_2_ availability is limited (i.e., the skin surface infected by the fungus)^23^. The genome of the fungal parasite *M. globosa*, the etiologic agent of specific skin diseases such as pityriasis versicolour, seborrhoeic dermatitis scalp and dandruff, contains a single gene encoding a *β*-CA (acronym MgCA). The enzyme showed an appreciable CO_2_ hydrase activity, with a k_cat_ value of 9.2 × 10^5^ s^−1^ and k_cat_/K_M_ value of 8.3 × 10^7^ M^−1^ s^−1^^24–30^. Primary sulphonamides resulted in excellent *in vitro* inhibitors (K_I_=63–174 nM)^24–30^. In contrast, other CA inhibitors such as inorganic anions, dithiocarbamates, monothiocarbamate, phosphonamidates, and phenols showed the K_I_ values in the µM range^26–28^^,^^31^. Subsequently, our groups investigated the biochemical properties and the sulphonamide inhibition profiles of the CA (MreCA) encoded by the genome of *M. restricta*^32^^,^^33^. This fungus is involved in starting the disequilibrium between the commensals *Cutibacterium acnes* (formerly named *Propionibacterium acnes*) and *Staphylococcus* sp*.*, both of which contribute to dandruff and seborrhoeic dermatitis symptoms^34^. MreCA showed a high catalytic activity for the hydration of CO_2_ into bicarbonate and protons (k_cat_ value = 1.06 × 10^6^ s^−1^ and k_cat_/K_M_ value = 1.07 × 10^8^ M^−1^s^−1^)^32^. Besides, primary sulphonamide inhibitors inhibited the enzyme with a K_I_ values <1.0 µM^33^.

In this article, we continue our research on fungal CAs, reporting the cloning, purification, and characterisation of the *β*-CA (MpaCA) from the pathogenic fungus *M. pachydermatis*, whose CA is homologous to MgCA and MreCA. We should stress that fungal CAs are proposed as potential biomolecules involved in the life cycle of the fungi. Thus, they could represent a new drug target for fighting the fungal infection as well as the drug resistance developed by Malassezia species or other fungi versus the drug compounds clinically used today.

## 2. Materials and methods

### 2.1. Bacterial strains, vectors, and chemicals

*Escherichia coli* DH5α cells (Agilent, USA) were used for initial cloning, while *E. coli* BL21 (DE3)pLysS cells (Agilent, Santa Clara, CA, USA) were utilised for the heterologous expression of the recombinant *M. pachydermatis* *β*-CA. The pET100/D-TOPO vector was purchased from Invitrogen (Carlsbad, CA) with the feature to express the recombinant protein as a fusion protein with a 6-histidine tag at the N-terminus. Luria Bertani Broth (LB), ampicillin, and other chemicals were obtained from Merck (Darmstadt, Germany).

### 2.2. Protein database screening

*M. pachydermatis* *β*-CA has been identified using the NCBI-BLASTP program, a sequence analysis tool specifically designed to search protein databases^35^^,^^36^. *M. globosa* *β*-CA was used as a query sequence to screen protein databases. The output file generated by the NCBI-BLASTP identified an amino acid sequence with the following accession number XP_017991749 (NCBI Reference Sequence), showing a high level of identity with respect to the homologous enzyme. The *Malassezia pachydermatis* *β*-CA was indicated with the acronym MpaCA.

### 2.3. Sequence analysis

The program MUSCLE, which was created for performing the multiple alignment of protein sequences, has been used to align the primary structure of all proteins here considered^37^.

### 2.4. Phylogenetic analysis

The program NGPhylogeny has been run to obtain a phylogenetic dendrogram, searching for the tree with the highest probability^38^.

### 2.5. Synthetic gene and cloning

The synthetic MpaCA gene was designed in our labs and produced by Life Technologies (Invitrogen, Carlsbad, CA), which is specialised in gene synthesis. The MpaCA gene contained NdeI and XhoI restrictions sites at the 5′- and 3′-ends, respectively, and four base-pair sequences (CACC) necessary for directional cloning at the corresponding 5′-end of the MpaCA gene. The synthetic MpaCA was ligated into the expression vector pET100/D-TOPO (Invitrogen, Carlsbad, CA) to form the expression vector pET100D-Topo/MpaCA, containing a nucleotide sequence encoding for a polypeptide with additional six histidines before the insertion point for facilitating the purification of the target protein. The MpaCA gene integrity and lack of errors in the ligation sites were confirmed by bidirectional automated sequencing.

### 2.6. Heterologous expression

Competent *E. coli* BL21 (DE3)pLysS (Agilent, Santa Clara, CA, USA) cells were transformed with pET100D-Topo/MpaCA. A single colony of transformed *E. coli* BL21 (DE3)pLysS was incubated overnight on a shaking incubator in 10 ml Luria-Bertani broth (LB) medium containing ampicillin (100 µg/mL), at 37 °C with constant agitation (200 rpm). The next day, 5 ml of cultured materials was removed and inoculated in 1 L of LB broth. The culture was grown at an OD_600nm_ value of 0.6 under vigorous shaking (200 rpm), at 37 °C. Isopropyl-β-D-thiogalactopyranoside (IPTG) was added to a final concentration of 1 mM, and 0.5 mM ZnSO_4_ was added after 30 min incubation for uptake in the expressed protein. The incubation period continued for additional 3 h, at 37 °C, with shaking at 200 rpm. Then, the bacterial suspension was tested and analysed on 12% SDS-PAGE to verify the overexpression of MpaCA. Sodium dodecyl sulphate SDS-polyacrylamide gel electrophoresis (SDS-PAGE) was performed as described by Laemmli using 12% gel^39^.

### 2.7. Enzyme purification

At 3-h post-induction, cells were harvested and disrupted by sonication at 4 °C. Following centrifugation, the supernatant was loaded onto HIS-Select HF Nickel Affinity Gel (Sigma-Aldrich, St. Louis, MO), equilibrated with 0.02 M phosphate buffer (pH 8.0) containing 0.01 M imidazole and 0.5 M KCl, at a flow rate of 1.0 ml/min. The recombinant MpaCA protein was eluted from the column with 0.02 M phosphate buffer (pH 8.0) containing 0.5 M KCl and 0.3 M imidazole at a flow rate of 1.0 ml/min. Active fractions (0.5 ml) were collected and combined to a total volume of 2.5 ml. Subsequently, they were dialysed, concentrated, and analysed by SDS-PAGE. The protein concentration of the purified recombinant enzyme was determined with a Bio-Rad protein assay based on the Bradford method^40^. At this stage of purification, the enzyme was at least 95% pure, and 1.0 mg of the total recombinant enzyme was obtained from 1 L of bacterial culture.

### 2.8. Enzyme protonography

For protonography, SDS-PAGE was performed as described by De Luca et al.^41^. Samples were mixed in a loading buffer without 2-mercaptoethanol, and they were not boiled to avoid protein denaturation. After electrophoresis, the gel was subject to protonography to detect the hydratase activity^41^.

### 2.9. Enzyme assay

An applied photophysics stopped-flow instrument has been used for assaying the CA catalysed CO_2_ hydration activity^42^. Phenol red (at a concentration of 0.2 mM) has been used as an indicator in a buffer containing 20 mM Tris (pH 8.3), 20 mM NaClO_4_ (for maintaining a constant ionic strength), measuring the absorbance maximum of 557 nm, and following the initial rate of the CA-catalysed CO_2_ hydration reactions for a period of 10–100 s. The CO_2_ concentrations values ranged from 1.7 to 17 mM during the determination of the kinetic parameters.

### 2.10. Inhibition assay with acetazolamide (AAZ)

For acetazolamide, at least six traces of the initial 5–10% of the reaction have been used for determining the initial velocity. The uncatalyzed rates were determined in the same manner and subtracted from the total observed rates. Stock solutions of inhibitor (10–50 mM) were prepared in distilled-deionized water, and dilutions up to 0.01 mM were done thereafter in the assay buffer. Inhibitor and enzyme solution were preincubated together for 15 min, at room temperature, to allow the formation of the E-I complex or for the eventual active site mediated hydrolysis of the inhibitor. As reported earlier, the inhibition constant values were obtained by non-linear least-squares methods using PRISM 3 and represents the mean from at least three different determinations^43^.

## 3. Results and discussion

**Figure 1.**
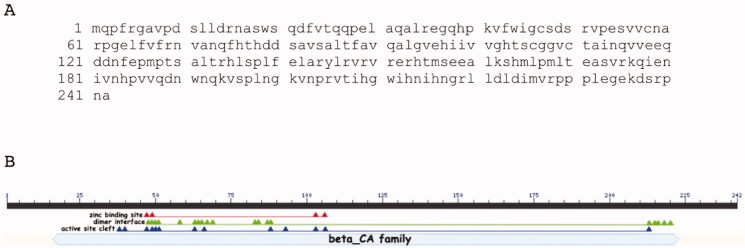
MpaCA amino acid sequence (A) and schematic representation (B) of the β-CA class consensus domains. Legend: lower letter case, MpaCA amino acid sequence; solid black line, polypeptide chain; solid light blue line, *β*-CA superfamily conserved domain; red triangles, conserved amino acids present at the enzyme’s zinc-binding site; green triangles, conserved amino acids present at the enzyme’s dimer interface; blue triangles, conserved amino acids present at the enzyme’s active site.

*M. pachydermatis* genome has a 726-bp gene region, encoding a CA polypeptide chain of 242 amino acid residues (Figure 1). Figure 1 was generated by the NCBI BlastP suite and showed that MpaCA contained all the consensus domains, which typify the β-CA class (Figure 1). Four amino acid residues of the zinc-binding site (C47, D49, H103, C106), thirteen residues of the active site cleft (Q38, P40, C47, D49, S50, R51, G63, F66, F88, L93, H103, C106, H213) and, as β-CAs are active only as dimers (or other multiple oligomers, such as tetramers or octamers),^44^^,^^45^ nineteen residues of the dimer interface (S48, D49, S50, R51, C58, G63, E64, L65, V67, R69, V83 S84, T87, F88, H213, I215, H216, G218, L220) have been mapped by the BlastP suite on MpaCA (Figure 1).

**Figure 2.**
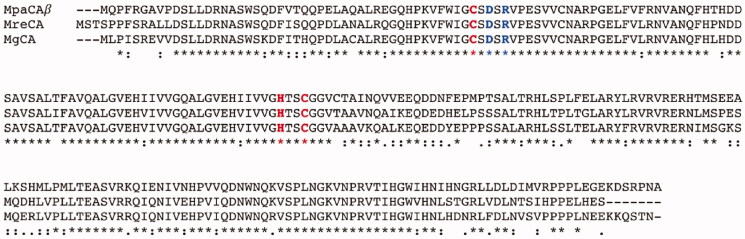
Multi-alignment of the *β*-CA polypeptide chains belonging to three Malassezia species, namely *M. pachydermatis*, *M. restricta,* and *M. globosa*. The multiple sequence alignment was obtained using the program MUSCLE. Legend: MpaCA, *β*-CA from *M. pachydermatis*; MreCA, *β*-CA from *M. restricta*; MgCA, *β*-CA from *M. globosa*; red amino acids, conserved residues present at the enzyme’s zinc-binding site; blue amino acids, conserved residues present at the enzyme’s catalytic dyad; asterisk indicates positions that have a single, fully conserved residue; colon indicates conservation between amino acids with strong chemico-physical properties; dot indicates conservation between amino acids with weak chemico-physical properties.

The encoded fungal enzyme was named MpaCA; it is homologous to the *β*-CAs previously identified by our group in the genome of *M. restricta* and *M. globosa*, which were annotated as MreCA and MgCA, respectively. To show the relevant degree of identity between these enzymes, we aligned MpaCA, MgCA and MreCA (Figure 2).

Figure 2 shows that the three enzymes have 173 fully conserved amino acids. In particular, MpaCA, MreCA, and MgCA have three residues (two cysteines and one histidine) totally conserved, which are involved in the catalytic mechanism of the enzyme, acting as zinc ligands, and the catalytic dyad (one aspartate and one arginine) near the first catalytic cysteine in the polypeptide chain (see Figure 2). Above-reported aspartate and arginine residues are involved in activating the zinc-coordinated water molecule responsible for nucleophilic attack to the substrate^46^.

**Figure 3.**
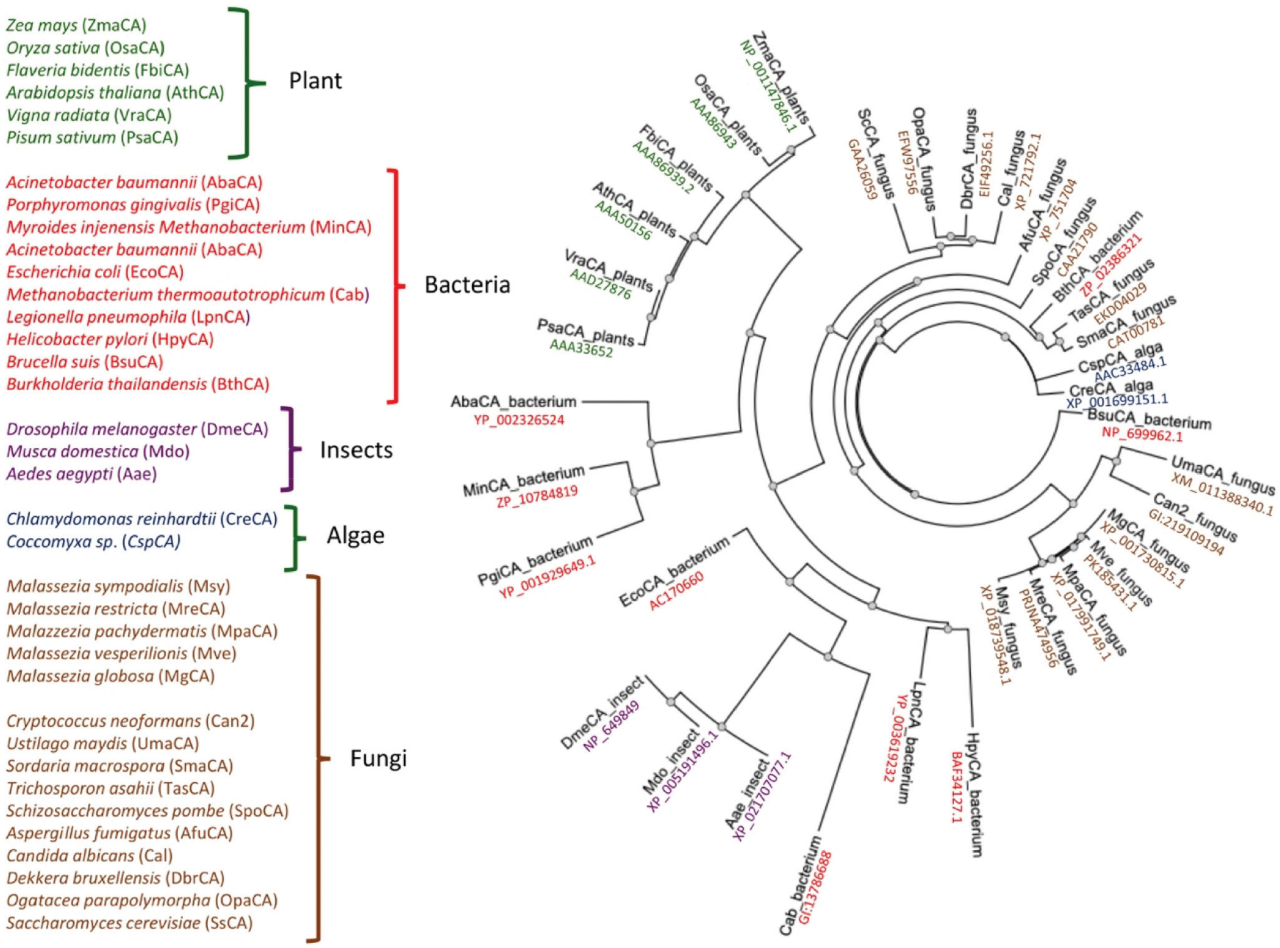
Radial dendrogram showing the evolutionary relationships of the *β*-CAs from various prokaryotic and eukaryotic species, such as plants, bacteria, insects, algae, and fungi. Species names and sequence acronyms are reported on the left of the figure. The accession number of the amino acid sequences used in the phylogenetic analysis are indicated below the sequence acronym shown in the radial dendrogram.

A most parsimonious phylogenetic tree was constructed to study the evolutionary links between the Malassezia β-CAs and similar enzymes identified in the genome of other fungi or different organisms belonging to other taxa, such as insects, plants, fungi, algae, and bacteria. As a result, a radial dendrogram has been generated, which is reported in Figure 3.

The Malassezia enzymes are closely related to each other and are phylogenetically very close to β-CAs from the pathogenic fungi *Ustilago maydis* and *Cryptococcus neoformans*. Intriguingly, the Malassezia β-CA cluster is well-separated from the other β-CAs identified in species of fungi different from Malassezia (Figure 3). We postulated that this is the result of a gene duplication event that occurred many millions of years ago throughout the history of the fungal *β*-CA gene, which separated the Malassezia β-CAs from those of other fungi, except *Cryptococcus neoformans* and *Ustilago maydis* (Figure 3).

**Figure 4.**
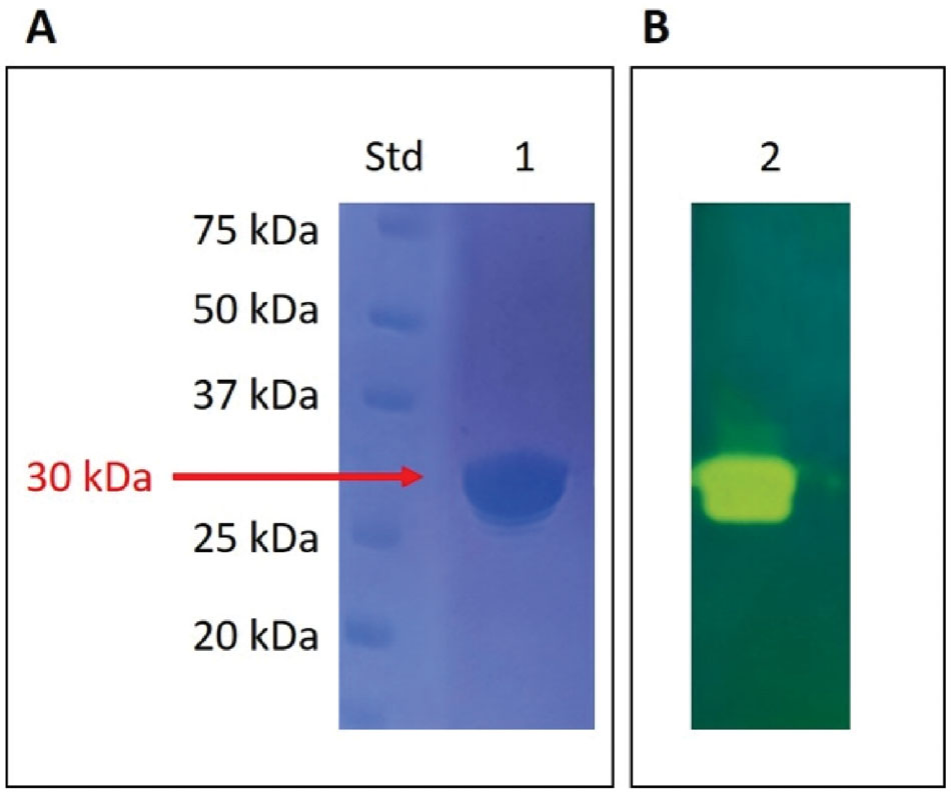
SDS-Page electropherogram (A) and protonogram (B). Legend: Lane Std, molecular markers, molecular mass values starting from the top: 75, 50, 37, 25, and 20 kDa; Lane 1: MpaCA band detected after staining Coomassie blue; Lane 2: MpaCA activity responsible for the reduction in pH from 8.2 to the transition point of the dye. The protein component corresponding to MpaCA is represented by the yellow band.

Our research on fungal *β*-CAs from Malassezia species prompted us to produce the recombinant MpaCA to compare its biochemical properties with those obtained for other two homologous Malassezia enzymes, MgCA and MreCA. The electropherogram developed by the SDS-PAGE of the fungal *β*-CA shows that MpaCA has been purified to homogeneity (Figure 4). The MpaCA monomer had an apparent molecular mass of about 30 kDa. The recombinant enzyme fused to the His-tag tail was expected to be 31 kDa.

The SDS–PAGE was also used to examine the MpaCA hydratase activity. In this case, MpaCA was loaded onto the polyacrylamide gel and subjected to protonography for detecting the ions (H^+^) caused by the MpaCA-catalysed conversion of CO_2_ into bicarbonate and protons. The pH variation due to the CO_2_ hydration reaction has been visualised as a yellow band in the protonogram, i.e., the electropherogram developed following the corresponding experimental protocol^41^^,^^46^. According to the results displayed in Figure 4, MpaCA resulted as an active enzyme migrating with a molecular mass of 30 kDa.

These results prompted us to investigate the MpaCA kinetic parameters using CO_2_ as substrate and the stopped-flow spectrometry as the technique allowing the study of fast reactions in solution. Table 1 shows a comparison of the kinetic parameters of recombinant MpaCA with those of MgCA and MreCA counterparts; the latter proteins were cloned as 6-histidine tag fusion polypeptides like MpaCA. For comparative purposes, we have also included two *β*-CAs from *Homo sapiens*, namely the isoform I (hCA I) and II (hCA II).

**Table 1.** Kinetic parameters of MpaCA compared with those of MgCA, MreCA and human isoenzyme hCA I and hCA II (α-class).

Organism	Enzyme Acronym	Class	k_cat_ (s^−1^)	K_M_ (mM)	k_cat_/K_M_ (M^−1^·s^−1^)	K_I_ (AAZ) (nM)
*Homo sapiens*	hCA I	α	2.0 × 10^5^	4.0	5.0 × 10^7^	250
	hCA II	α	1.4 × 10^6^	9.3	1.5 × 10^8^	12
*Malassezia pachydermatis*	MpaCA	*β*	3.8 × 10^5^	39.7	9.7 × 10^6^	623
*Malassezia restricta*	MreCA	*β*	1.06 × 10^6^	10.1	1.07 × 10^8^	51
*Malassezia globosa*	MgCA	*β*	9.2 × 10^5^	11.1	8.3 × 10^7^	74,000

The β-class enzymes were tested for the CO_2_ hydration reaction in 20 mM Tris buffer pH 8.3 and 20 mM NaClO_4_, at 25 °C.

Reported mean values are from 3 different assays performed by the stopped-flow technique; errors were in the range of ±5–10% of the reported values (data not shown).

MpaCA showed a significant catalytic activity, with a k_cat_ value of 3.8 × 10^5^ s^−1^ and a k_cat_/K_M_ value of 9.7 10^6^ M^−1^ s^−1^. The data reported in Table 1 demonstrate that MreCA has a catalytic activity (k_cat_ value of 1.06 × 10^6^ s^−1^ and k_cat_/K_M_ value of 1.07 × 10^8^ M^−1^ s^−1^) higher than that of MpaCA and MgCA, being in the same order of the human isoform hCA II, which is considered among the fastest CA known so far. Although MreCA, MpaCA, and MgCA have 173 fully conserved amino acids within a 242 amino acids-long polypeptide chain, they exhibited a pronounced difference in their inhibition behaviour with respect to the classical primary sulphonamide inhibitor acetazolamide (AAZ). MpaCA displayed an inhibition constant (K_I_) value of 623 nM, which resulted only 2.5 times higher than that of hCA I (K_I_ value= 250 nM) (Table 1). Instead, the MreCA activity was highly inhibited by AAZ, with an inhibition constant of 50.7 nM, while MgCA was slightly sensitive to AAZ inhibition (K_I_ value= 74,000 nM).

These findings could pave the way for developing highly selective drugs that do not interfere with human skin integrity by inhibiting the *β*-CAs encoded by healthy scalp microbes, which probably are well inhibited by AAZ as the other bacterial -CAs, and avoiding interference with human CAs since mammals only contain *α*-CAs.

## 4. Conclusions

The ability to enter a host, evade host defences, grow in a host environment, counteract host immune responses, assimilate iron and nutrients from the environment, and perceive environmental change are all prerequisites for microorganism pathogenicity. Many enzymes aid the pathogenicity of the microbes. Among them, it is possible to mention proteases, neuraminidases, phospholipases, and ureases^47^. Recently, a new superfamily of metalloenzymes, namely CAs, has been identified as biomolecules playing a pivotal role in microbial virulence and pathogenicity^18^^,^^20^^,^^48–51^. The CO_2_-sensing, triggered by HCO_3_^−^ produced in a CA-dependent manner, is an essential modulator of fungal metabolism and pathogenicity. In this context, we have reported here the cloning, purification, and initial characterisation of MpaCA encoded from the genome of the pathogenic fungus *M. pachydermatis*, which is the aetiological agent of otitis externa and seborrhoeic dermatitis in dogs, cats, and other wild animals^3^. The recombinant MpaCA, prepared as 6xHis-tag fusion protein, was efficiently expressed and purified by affinity chromatography. MpaCA showed a significant catalytic activity for the hydration of CO_2_ into bicarbonate and protons, with the following kinetic parameters: k_cat_ value of 3.8 × 10^5^ s^−1^ and k_cat_/K_M_ value of 9.7 × 10^6^ M^−1^ s^−1^. The enzyme is also sensitive to inhibition by the classical sulphonamide inhibitor acetazolamide (K_I_ value of 50.7 nM). Intriguingly, although Malassezia *β*-CAs resulted phylogenetically close to each other, they showed substantial differences in their inhibition with AAZ as well as in their catalytic constants; in particular, the k_cat_ value of MreCA was an order higher than those of MpaCA and MgCA. Further X-ray crystallographic studies on MpaCA, MreCA, and MgCA with classical CA inhibitors will be helpful in better understanding the inhibitory behaviour of these and other fungal *β*-CAs, whose *in vivo* inhibition might be essential for fighting fungal diseases and the phenomenon of the drug resistance.
